# The Potential Role of Genetic Markers in Talent Identification and Athlete Assessment in Elite Sport

**DOI:** 10.3390/sports6030088

**Published:** 2018-08-30

**Authors:** Ysabel Jacob, Tania Spiteri, Nicolas H. Hart, Ryan S. Anderton

**Affiliations:** 1 School of Health Sciences, University of Notre Dame Australia, Fremantle 6160, Australia; yzzyjacob@gmail.com (Y.J.); t.spiteri@ecu.edu.au (T.S.); 2 School of Medical and Health Sciences, Edith Cowan University, Joondalup 6027, Australia; n.hart@ecu.edu.au; 3 Institute for Health Research, University of Notre Dame Australia, Fremantle 6160, Australia; 4 Exercise Medicine Research Institute, Edith Cowan University, Joondalup 6027, Australia; 5 Centre for Neuromuscular and Neurological Disorders, University of Western Australia, Nedlands 6009, Australia; 6 Perron Institute for Neurological and Translational Science, Nedlands 6009, Australia

**Keywords:** performance, ACE, ACTN3, talent identification, genetic polymorphism

## Abstract

In elite sporting codes, the identification and promotion of future athletes into specialised talent pathways is heavily reliant upon objective physical, technical, and tactical characteristics, in addition to subjective coach assessments. Despite the availability of a plethora of assessments, the dependence on subjective forms of identification remain commonplace in most sporting codes. More recently, genetic markers, including several single nucleotide polymorphisms (SNPs), have been correlated with enhanced aerobic capacity, strength, and an overall increase in athletic ability. In this review, we discuss the effects of a number of candidate genes on athletic performance, across single-skilled and multifaceted sporting codes, and propose additional markers for the identification of motor skill acquisition and learning. While displaying some inconsistencies, both the ACE and ACTN3 polymorphisms appear to be more prevalent in strength and endurance sporting teams, and have been found to correlate to physical assessments. More recently, a number of polymorphisms reportedly correlating to athlete performance have gained attention, however inconsistent research design and varying sports make it difficult to ascertain the relevance to the wider sporting population. In elucidating the role of genetic markers in athleticism, existing talent identification protocols may significantly improve—and ultimately enable—targeted resourcing in junior talent pathways.

## 1. Introduction

It is commonly discussed in the talent identification (TID) circles whether sporting talent is “born or bred”. In a sporting context, there is a limit to the improvement an athletes’ performance can make from practice alone; inferring that ability and skill are, at least, partially inherited qualities [1]. Short of there being a single gene responsible for sporting success, it is estimated that the human genome has 10 million different single nucleotide polymorphisms (SNPs), with dozens currently linked to sporting performance and sporting success across various sporting codes [2].

The ability to maximise athlete performance and athlete potential in elite environments is constantly at the forefront of sport science. New technology and training methods are continually being tested and developed to enhance performance and elevate athletes to an elite level status. When considering athletic performance, numerous generic and sport-specific assessments are performed to identify sporting talent and to provide an indication of current athletic ability. The notable increased inclusion of skill assessments in current athletic screening and testing batteries allows the quantification of technical and tactical attributes of an athlete to determine how one may perform in competition. Specifically, the utilisation of sport-specific skill assessments, including match day statistics, are commonly used for determining player performance in Australian Football (AF), basketball, soccer, and rugby union [3,4,5,6,7,8,9,10]. These assessments allow for an evaluation on player skill performance and match day impact.

Currently, there are several methods of determining individual player achievement within a sport: team selection, career success, coach perception, and match performance within a season [11,12]. In the context of AF, athletic field tests—such as the vertical jump, 20-metre sprint, and endurance time trials—are often used in the talent identification process to acknowledge athletes with the potential to succeed in a given sport [13]. These tests reveal indications of physical capabilities that may enhance or hinder an athlete’s ability to perform within a given sport or position within a sport. Similarly, skill-based assessments have been introduced as an additional element, as a method to rank athletes on their technical abilities [14]. As these tests are often performed within a testing battery during events such as draft combines, the results from these tests give information on the skill level of an athlete, and therefore an indication of the individual work required to elevate their abilities to an elite level.

Common genetic polymorphisms (natural variations in genetic sequences) account for the variability in the expression of several key genes important in the regulation of physiological processes. Among these, single nucleotide polymorphisms (SNPs) have gained significant attention as contributors of behavioural, physiological, and cognitive variability [15]. Owing to the diverse array of desirable physical attributes in sport, several polymorphisms within the human genome have previously been highlighted. For example, the angiotensin-converting enzyme (*ACE*) and alpha-actinin-3 (*ACTN3*) genes have both been linked with elite levels of athletic performance in endurance and/or strength/power dominant sports; including long distance running and swimming, road cycling, rowing, cross-country skiing, triathlons, sprinting, volleyball, track and field jumping and throwing, weightlifting, ice hockey, wrestling, and figure skating [16,17,18,19,20]. In addition, specific homozygous genotypes in the peroxisome proliferator-activated receptor gamma co-activator 1-alpha (*PPARGC1A*) gene and the beta-adrenergic receptors 1/2/3 (*ADRB 1/2/3*) have been linked with higher maximal oxygen uptake (VO_2_max), better endurance performance and more favourable body mass index in long-distance runners, long-distance swimmers road cyclists, cross-country skiers, triathletes, sprinters, sprint swimmers, weight and power lifters, track and field jumpers and throwers, boxers, and kayakers [21,22,23].

Despite the intriguing nature of these studies, there has been no strong candidate genes associated with performance in skill execution within sport. Polymorphisms within the brain-derived neurotropic factor (*BDNF*), dopamine D2 receptor (*DRD2*), and catechol-O-methyltransferase (*COMT*) genes have all been associated with motor control and/or learning [24,25]. However, none of these genes have previously been investigated in the context of sport-specific skill analysis. In AF, using standard skill performance assessments, a relationship between player genotype and performance has been identified [5]. However, the genetic implication of these tests has only been previously investigated once, in a small cohort of sub-elite AF players, while match performance has not been investigated in a genetic capacity at all.

## 2. Talent Detection and Identification

All traditional athlete development pathways share a common goal, to identify and develop individuals with the greatest long-term potential for success in elite competition [13]. The common development process of many sporting codes reflects the model proposed by Williams and Reilly [26], which involves the process of athlete detection, identification, selection, and development. In this model, athletes are selected to participate in development programs based on detection (the process of unearthing potential elite performers not currently participating in the sport in question) or identification (recognising those currently in the pathway with the potential to excel) outcomes. Athlete detection and identification is critical to the athlete development process, as they typically guide the initial, and subsequent, selection of athletes into development programs.

Assessments utilised in the identification and detection process are typically considered sport specific. For example, the testing battery used at national draft combines of the Australian Football League (AFL), National Football League (NFL), and National Basketball Association (NBA) may be considered highly specific to each of the particular sports. Each sport adopts performance tests that are considered to best represent the physical demands of that particular sport [13]. Due to the multi-disciplinary nature of most team sports, testing often examines a range of physical performance measures—such as aerobic capacity, anaerobic power, and technical skill—with results used to guide higher or elite level selection [27,28,29]. However, while there are test that are specific to each sport, usually skill specific, these tests are performed in closed-environment settings and do not have the ability to assessment an athlete’s decision making skills or execute skill under pressure. The lack of testing in open-environment is a flaw in current TID testing batteries.

## 3. Measurements of Sporting Performance

Prior to discussing gene candidates and their potential relation to athlete performance, skill performance, and match performance, it is first important to identify and describe the current assessments commonly utilised to measure these athletic performance domains, and so too the qualities they measure.

### 3.1. Measurements of Athletic Performance

#### 3.1.1. Aerobic Assessment

An athlete’s ability to recover between bouts of high intensity effort is linked to their aerobic capacity [30,31]. Aerobic capacity is most accurately assessed as a measure of maximal oxygen uptake (VO_2_max) [32,33], however true VO_2_max testing in team sport environments is uncommon due to specialist equipment requirements and cost. Due to these limitations, predicted VO_2_max measures are more commonly used. Many indirect measures exist, with most assessments being either timed continuous runs, or interval based tests [34,35,36,37]. Recently, there has been a transition towards the Yo-Yo Intermittent and 30:15 Intermittent Recovery tests, as the recovery periods dispersed within the tests are more sport specific. A version of the Yo-Yo test, the Yo-Yo IR2, has been shown to have a strong relationship with high-speed running and match performance (measured by possessions) during an AF game [8].

#### 3.1.2. Strength and Power Assessments

In many sporting codes, the ability of an athlete to jump higher than their opponent may put them at a distinct competitive advantage. As such, the vertical jump (VJ) test is commonly included as a TID assessment, as a measure of explosive anaerobic power of the lower limbs [38,39,40]. To assess an athlete’s VJ capability, there are typically two different tests performed, a stationary countermovement jump and a dynamic countermovement jump. In both instances, the aim of the test is for the athlete to attain maximum vertical displacement. The dynamic vertical jump is included in the testing barrage of many sports, and is considered a more reflective test for power assessment in team sports. However, while the VJ is a good measure of anaerobic power, it does not often relate to success in Australian Football [11].

The peak force generated during the isometric mid-thigh pull (IMTP) strength test has also been associated with VJ. The peak force was significantly related to countermovement and squat jump performance in junior male and female surfers [41]. The IMTP has been used to assess collegiate level American Football players and wrestlers, and was found to be an effectual and fast technique of measuring isometric strength [42,43]. When tested in female Olympic weightlifters, a near perfect relationship between peak force during the IMTP and VJ peak power was seen [44]. Similar results were seen in former colligate level male weightlifters, as there was a strong correlation between isometric peak force and VJ performance [45]. The IMTP can also be used to monitor athletes, such as weightlifters, as it delivers information of strength and explosiveness [46]; key areas in many sports including AF.

#### 3.1.3. Acceleration and Maximal Linear Sprinting Speed

To assess an athlete’s sprint capacity, maximum acceleration, and speed, and the ability to perform repeated high-speed efforts are commonly used in talent pathways [47,48]. Often, acceleration and maximum speed are assessed concurrently, with timing gates used to assess an athlete’s linear speed often over 20–60 m [29,40,49]. In Australian Football (AF), the 20-m sprint has been shown to be predictive of selection into underage representative and professional senior squads [27], and AFL success [11]. Such an assessment is a good indicator of acceleration [27,50] and often provides important performance information for recruiters in power-based sports, such as American football [51].

#### 3.1.4. Muscle Fibre Composition

The structure and composition of skeletal muscle fibres has the potential to be used as a predictive measure for athletic performance. For example, slow-twitch fibres (type I) are associated with endurance events, while fast-twitch fibres (type II) are linked to short, explosive events. Type I fibres have a higher aerobic capacity and myoglobin stores, allowing for greater blood flow and improved oxygen delivery to the muscle [52]. In contrast, type II fibres breakdown ATP rapidly, resulting in a fast muscle shortening cycle, and the production of more force [53,54]. Fast twitch fibres have an abundance of glycolytic enzymes, allowing for a large anaerobic capacity [55]. As a result, type II fibres are more fatigable than type I fibres. 

Another method for muscle fibre assessment involves the measurement of muscle carnosine, a muscle metabolite that cannot be easily influenced by environment, training, or diet [56,57]. Interestingly, fast-twitch fibres can have twice the amount of muscle carnosine than slow-twitch [57]. In athletes, levels of muscle carnosine are approximately 30% higher in sprinters and short-event athletes, and approximately 20% lower in endurance event athletes, compared to the control group [57]. The relative amounts of alpha-actin-3 and alpha-actin-2 can also influence muscle fibre composition [58]. Alpha-actin-3 (fast-twitch) and alpha-actin-2 (slow-twitch) have the ability to influence fibre characteristics in individual muscle [58]. The alpha-actin genotype can affect fibre-type arrangement, metabolic profile, and contractile properties [58].

### 3.2. Measurements of Skill Performance

The sport specific assessment of skill is an essential and commonly examined component of the TID process [14,39,40,59]. For example, in table tennis, eye-hand coordination is the first skill assessment in the TID process [60]. Similarly, there have also been strong associations between motor coordination and status level in female volleyball players, a sport where motor coordination is an indicator of an athlete’s potential [61]. However, the assessment of skill is often a subjective evaluation and therefore has an area of biased opinion [62]. Frequently, skill assessment is rated on skill-specific criteria and is graded on a Likert scale, but due to the subjectivity of the observers making the assessment, the reliability can be questionable. It must also be stated that some sport-specific skills, such as tackling in rugby league, are more difficult to assess reliably, compared to other skills [62].

### 3.3. Measurements of Match Performance

Match performance is a measurement of contributions to a game and is reported as game day statistics. Individually, methods of determining player achievement can be completed subjectively via umpire or coach votes, or objectively using match-based statistics [12]. Using match statistics, such as handballs and kicks, an objective measurement of match performance can be calculated for AF players [11,50]. Match statistics are accumulated in AF by a commercial analytics company (Champion Data, South Bank, Australia) with reports of 99% accuracy [63] and have been previously used in other studies involving AF [8,64]. Direct game involvement (DGI) can be used as a sum of handball, kick, mark, and tackle statistics is a game of AF, and DGI per minute (DGI/min) can be used as a measurement of game impact [12].

Often, talent identification and pre-season testing is used as an indicator of potential match performance and fitness. Aerobic time trials, which are often apart of such testing batteries and pre-season fitness measures, have previously been associated with improved match performance in sub-elite AF players [12]. In lower body power assessments, the vertical jump does not correlate with success in AF [11]; however, the 20-m sprint shows moderate positive correlations [11,27]. Coaches and high performance managers may desire a more accurate and efficient method of gauging physical capacities during testing and pre-season training, which opens the opportunity for the investigation of genetic profiles in certain sports.

## 4. Contribution of Genetic Variation to Athlete Strength, Power, and Endurance

The question is often posed, what makes an athlete? External influences such as early-life exposure, social-economic status, and training history influence an individual’s ability to succeed in sport. However, a player’s genetics may play an important role in determining sporting achievement, as athleticism, like many other individual characteristics, is, at least, a partially inherited trait [1]. There are allelic variants within genes that have been identified as predisposing individuals to elite endurance or elite power. The *ACE* and *ACTN3* genes both have one homozygous variation that predisposes individuals to power and another to endurance [18,65,66,67,68,69]. While the *ADRB 1/2/3* and *PPARGC1A* genes that influence endurance performance, one homozygous variation positively impacts endurance performance and the other negatively impacts endurance performance [21,22,23,70,71,72,73,74]. Besides athletic traits—such as endurance, strength, and power—other traits such as skill acquisition and development affect a person’s success in sport, especially team sports such as AF. There is evidence from several genes (*BDNF*, *DRD2*, *COMT*) that show they impact motor coordination and/or development [24,25,75].

### 4.1. The Role of ACE

The human *ACE* gene is an important part of the renin-angiotensin-aldosterone system (RAS). This system is involved in circulatory homeostasis [76], skeletal muscle growth [77] and cardiovascular function [78]. The *ACE* gene is located on chromosome 17 and has an insertion/deletion polymorphism of 287 base pairs, resulting from an Alu repeat sequence, shown to insert within intron 16 [79]. This produces three well-documented *ACE* insertion/deletion genotypes; II homozygote, DD homozygote, and ID heterozygote. Previous studies have shown the *ACE* polymorphism to be correlated with cardiovascular disease risk and complications [80], proliferative diabetic retinopathy [81], Alzheimer’s disease [82], and acute macular degeneration [83]. The frequency of the *ACE* I/D genotype shows variation amongst ethnic populations [84,85], and also correlates with athletic traits in a number of elite sporting codes. However, in simple movements such as a squat jump or countermovement jump, neither homozygous *ACE* genotype (DD or II) has shown correlation to performance [86].

The *ACE* insertion allele indicates the presence of an *Alu* repeat sequence within the gene. The homozygous insertion allele (I) is associated with lower plasma levels of ACE enzyme, and has been linked to the onset and progression of Alzheimer’s disease [87]. In a sporting context, the frequency of I alleles appears to increase with race distance in studies of elite Caucasian runners [18] and swimmers [68]. Therefore, the insertion *ACE* allele appears to be an advantageous genotype for endurance sports. Athletes with this allele tend to demonstrate an increased aerobic capacity [79,88] and better performance in a range of endurance sports [89]. However, a smaller number of contradictory studies have also demonstrated no relationship between the I allele and endurance athletes [90] or maximal oxygen consumption (VO_2_max) [89], a measure of aerobic capacity. Possible reasons for these contradictions include diverse group of athletes with a small percentage of endurance athletes, the examination of elite and sub-elite athletes, and environmental factors [88]. Orysiak, Zmijewski, Klusiewicz, Kaliszewski, Malczewska-Lenczowska, Gajewski, and Pokrywka [88] even suggested that the *ACE* gene has a minor role in endurance performance, implying that the *ACE* polymorphism has a greater relationship with strength and power, and the deletion allele. 

The deletion allele of the *ACE* gene has commonly been studied in association with sporting codes dependent on strength and/or power. This particular allele is associated with circulating plasma levels of *ACE*, with a DD genotype resulting in significantly higher levels of *ACE* compared to a II genotype [91]. Elevated levels of *ACE* can contribute to ischaemic heart disease and other complications arising from increased activation of the angiotensin system [92]. However, the deletion genotype (DD) has also been associated with increased musculoskeletal fitness [93] in strength-related individual sports. For example, European Caucasian elite short distance swimmers [18,69], and track and field [67] athletes, were found to have increased D alleles compared to controls. In contrast, a population of Lithuanian soccer players [78] and South East Asian rugby players [79] were found to have significantly lower proportions of the D allele. This is surprising, considering that both sporting codes have elements of strength and power, suggesting ethnicity and additional factors may also be at play. Similar studies have also revealed no correlation between *ACE* I/D alleles and individual [67,94,95] and team [95] sports (Table 1). More recently, a multinational study investigating the *ACE* I/D alleles in Caucasian endurance athletes failed to identify a correlation between genotype and performance time [96].

### 4.2. The Role of ACTN3

A member of the actin family, actini-alpha-3 (ACTN3) is a protein highly expressed in muscle tissue, which functions to crosslink actin filaments in fast-twitch (type II) skeletal muscle fibres [97]. Expression in glycolytic skeletal muscle allows this protein to contribute towards powerful muscle by coordinating fast-twitch muscle contractions [97]. The *ACTN3* gene, coding for this protein, has been thrust into the forefront of genetic studies following the identification of a nonsense polymorphism at position 577 (rs1815739), which significantly affects ACTN3 protein levels due to a premature stop codon within the *ACTN3* gene [97]. The most common nucleotide at position 577 is a cytosine (C allele) encoding an arginine amino acid (arginine = R), with the alternative T allele encoding a stop codon (stop = X). Therefore, within the literature, the CC genotype is referred to as RR, whereas the TT genotype is often called XX (referred to herein as R577X). Interestingly, over a billion people worldwide are believed to carry the homozygous absent XX genotype and be deficient in this protein [97,98]. Studies examining the effect of an *ACTN3* knock out in mice show alpha-actin 2 levels increase, resulting in a transition of fibre type towards fatigue resistance, as a compensatory mechanism for the absence of functional ACTN3 protein [58]. Similar to in vivo studies, athletes with an XX genotype show an increased tendency to perform endurance sports, as shown by an increased frequency of this particular allele (Table 1).

There is currently a strong association between the homozygous R allele of *ACTN3* and strength/power athletic phenotypes [16,97,99,100,101]. Furthermore, there has also been evidence that a higher proportion of slow-twitch muscle fibres are related to the X allele genotype and elite endurance status [17,98,102]. In team sports, a cohort of Brazilian soccer players with the RR genotype performed significantly better at short distance sprints and jump tests compared to athletes with the RX and XX genotypes [103].

### 4.3. Additional Candidate Genes Implicated in Strength and Power

A number of additional genes have been identified as being strong indicators of athletic status (see Table 1). The majority of these genes encode proteins that are associated with muscle tissue or cardiovascular function [104]. The angiotensin II receptor type 2 (*AGTR2)* gene, associated with the RAS system, has been implicated in power sports due to its association with fast-twitch muscle fibre composition [77]. A polymorphism in this gene (rs699) has been found to occur more frequently in females (A allele) than males (C allele). Interestingly, the A allele correlates with selection to strength and power sports, when compared to control or C allele groups [77].

Similarly, a number of polymorphisms relating to genes responsible for oxygen delivery have been identified as predictors for strength and power performance. The two variations in the endothelial PAS domain protein 1 (*EPAS1*) gene, the hypoxia inducible factor 1 A (*HIF1A*) and the nitric oxide synthase 3 (*NOS3*) genes, have shown an association with sprint and power sports, when compared to control groups [105,106,107]. Within a group of Polish and Russian athletes, two homozygous genotypes of the *EPAS1* variants were found to be under-represented in sprint and power athletes [107]. While the CC genotype of the *HIF1A* gene is more frequent in wrestlers and weightlifters [106] and the T allele of the NOS3 gene has been shown to be associated with, therefore beneficial to, power sports [105].

### 4.4. Additional Candidate Genes Implicated in Endurance and Aerobic Capacity

Despite a heavy focus on the role of the *ACE* insertion/deletion polymorphism, there have been a number of recent genes implicated in aerobic/endurance performance (see Table 1). The *ADRB 1/2/3* genes are an example of this, and function to encode G-coupled receptors in cardiac and adipose tissue, regulating cardiac function and metabolism [73]. Specifically, a single base variation in *ADRB1* (rs1801253) can drastically increase VO_2_max, exercise time and endurance (C allele), or cause a decrease in VO_2_max (G allele) [74]. Similarly, the G allele in *ADRB2* (rs1042713) is strongly correlated with increased BMI and decreased VO_2_max [72]. Finally, a study on Spanish athletes showed a rare C allele in *ADRB3* (rs4994) strongly correlated with elite endurance performance [23], making this an ideal predictive marker.

The *PPARGC1A* gene plays an important role in metabolism. *PPARGC1A* functions as an activator of oxidative phosphorylation genes, ultimately controlling glucose and lipid metabolism [70,71]. Further, it has been linked to skeletal muscle fibre formation, and the determination of fibre type [71]. A single base variation (rs8192678) in different populations can correlate to different outcomes. For example, studies show that European men homozygous for the A allele (AA genotype) have an extremely strong correlation with higher VO_2_max and endurance. In contrast, Chinese men that are homozygous for the G allele (GG genotype) show a strong association with higher VO_2_max and endurance [21,22].

## 5. Genetic Links to Ability and Skill in Sport

### 5.1. BDNF Polymorphism and Motor Skill Acquisition

Brain-derived neurotropic factor (BDNF) protein has an effect on vascular and neuronal growth, as well as brain, spinal cord, skeletal muscle development and regeneration [119,120,121,122,123]. Structural and functional changes can occur due to different SNPs in the *BDNF* gene [124]. The binding and internalisation of the BDNF protein can affect axonal path finding [125], differentiation, neuronal survival [126], formation and conservation of late-phase potentiation [127], hippocampus neuronal death defence [128], and dendritic trafficking regulation [129].

The *BDNF* Val66Met polymorphism (rs6265), found on codon 66 [75], is known to impact on the ability of humans and mice to develop motor skills [24]. Fritsch, Reis, Martinowich, Schambra, Ji, Cohen, and Lu [24] found that BDNF levels are elevated during motor skill training, when participants completed a sequential visual isometric pinch task. This could be due to the secretion of BDNF needed to increase synaptic plasticity in the motor cortex of the brain [24]. Corticospinal output of BDNF encoded by the AA genotype increased after motor training, while AG and GG genotypes showed reverse effects [130]. This suggests that the effect of the polymorphism does not occur in the basal state, but due to increased neural activity as a behavioural response [130]. The Val66Met polymorphism is also related with short term reduced motor learning and changed motor cortex plasticity [75]. For example, AG carriers exhibit poorer motor learning in simple motor tasks, such as a serial reaction time task, compared to AA genotypes [75].

### 5.2. Dopamine Receptors and Procedural Learning of Complex Skills

Dopamine polymorphisms are associated with many neurological and mental disorders that are characterised by changes in cognitive and emotional processes [25]. Huertas, Buhler, Echeverry-Alzate, Gimenez, and Lopez-Moreno [25] found a connection between a SNP in the *DRD2* gene (rs1800497), situated in exon 7, which affects mirror drawing, a visual-motor task that requires procedural learning. From this study, C allele and T allele carriers showed a similar ability at the commencement of the task, however C allele carriers learnt more as the task continued [25]. The results were unexpected as C allele carriers are related to low striatal DRD2 availability [131] and high DRD2 binding potential within the thalamus and cortex [132]. However, there are two possible explanations for this. The low striatal density, linked with the C allele genotype, may cause a higher affinity to compensate by an optimisation of G-protein-coupled signalling, leading to more effective learning [25]. Another explanation is that in the study, both CC and TT genotypes showed an increased heart rate during the task; stress has been showed to increase striatal dopamine release [133]. The stress produced during the task may have caused compensation in the low striatal density, providing optimal learning circumstances [25].

Catechol-O-methyltransferase (COMT) is an enzyme that promotes the breakdown of dopamine, a process essential for cognitive function [134]. A *COMT* polymorphism at position 158 (Val158Met), resulting in either an A or G allele (rs4680), has been associated with motor control, and linked with the expression of *DRD2* [135]. Individuals homozygous for the A allele (Met) have reduced COMT enzyme activity, resulting in increased dopamine activity, compared with G carriers (Val) [135]. Furthermore, pairing both homozygous AA *COMT* and CC *DRD2* genotypes can allow for higher rates of motor learning [135]. While these genes have been linked to motor learning and coordination, it has not been investigated whether they affect sport-specific skill acquisition.

## 6. Ethical Considerations

It is important to consider the ethical issues that could arise from the genetic testing of potential athletes. From a public health perspective, medical genotyping is seen as presumptuous, divisive, and discriminatory [136]. Therefore, in a sporting context, the suggestion that athletes should be left out of elite training pathways based solely on their genome would likely be considered by many as unethical [137]. However, including a junior athlete in a development program based on their genotype is a more inclusive measure of TID and could allow for late-developers to receive appropriate coaching while they mature [138]. This would allow for fair selection once all the athletes have reached the same maturity as the early-developers would not have been put at an advantage based on anthropometric and physical testing from adolescent years. The genetic screening should not be used as a measure of complete athletic potential, but as a predictive tool in athlete potential [138]. Other ethical concerns that arise from the notion of genetic testing for TID purposes are the restricting of participation options, early specialisation risks, and potentially missing out on other opportunities that could provide enjoyment [139]. However, what genetic testing can be used for is guiding interventions that could reduce injury and improve an individual’s health [137,140].

## 7. Conclusions

Current TID processes have been utilised for years with basic practises having been expanded on to include sport-specific tasks, involving skill. With modern technology, there is potential to examine an athlete’s genetic makeup and investigate whether a particular genotype can contribute to athletic performance. The *ACE* and *ACTN3* genes have shown strong potential to predict athletic prowess in both endurance or power and strength-based sports, while numerous other genes have shown associations with one or the other. Even more exciting is the possibility to prophesise motor control and development in relation to skill acquisition and performance in sport. Variants in the *BDNF*, *DRD2*, and *COMT* genes are possible candidates for future research aiming to investigate such possibilities in different sports. However, it must be stressed that the identification of athletic talent is unlikely to be the result of a small number of genetic variants, rather a complex combination of a large number and pattern of expressed genes, as well as a number of environmental conditions. 

The ability to develop training programs custom made for athletes based on their known areas of need has very plausible implications. Most coaches have used the phrase “practise makes perfect” and many coaches and sport scientists will say that training time is limited for a multitude of reasons.

Research into this area can be used to determine if a particular genotype allows for athletes to better perform and react to certain training stimuli, allowing for a more well-rounded view on training specification. Further research conducted should therefore investigate how a player’s genotype may contribute to training outcomes and adaptations. A major draw to prospective athletes to a given sporting club, or institute, is their ability to cater and individualise training programs to the individual athlete. Introducing genetic testing to these programs will allow athlete specific training regimes to improve athletic, skill, and match performance.

## Figures and Tables

**Table 1:** Previous studies assessing the role of genes in determining athletic performance

Gene	Sport/Discipline	Outcome of Study	Reference
*ACE*	Basketball	Higher proportion of I allele frequency than D allele amongst athletes	[90]
	Cross country skiing	No significance reported	[67]
	Cycling	Significantly higher I allele frequency among long-distance Spanish, male, elite cyclists	[65]
		Higher proportion of D allele frequency than I allele amongst athletes	[90]
	Endurance sports	Increased frequency of DD genotype in endurance athletes compared to power athletes	[84]
	Handball	Significantly higher I allele frequency among Spanish, male, elite handball players (national team)	[65]
	Power sports	Decreased frequency of DD genotype in elite Korean power athletes compared to a control group	[85]
	Running (long distance)	Increasing I allele frequency with increasing race distance in elite British and Spanish runners	[18,65]
		No significance reported	[96]
	Running (short distance)	Increased DD genotype and D allele frequency in sprinters	[18,19]
		No significance reported	[94,95]
	Rhythmic gymnastics	D allele was more frequent in elite level gymnastics compared to sub-elite athletes and controls	[108]
	Soccer	Significantly lower DD, greater ID genotype in Lithuanian professional soccer players	[78]
		Significantly higher frequency of ID genotype and lower frequency of II genotype in soccer players compared to endurance runners	[109]
	Swimming	Significant association between the DD genotype and elite, short distance swimmers	[69,110]
		Significantly greater I allele in middle distance Russian swimmers. Increasing I allele frequency with increasing race distance in elite long distance swimmers	[67,68]
		No significance reported	[95]
	Volleyball	Higher proportion of I allele frequency than D allele amongst athletes	[90]
		No significance reported	[95]
	Weightlifting	Equal distribution of D and I alleles amongst athletes	[90]
*ACTN3*	Endurance sports	No significance reported	[96,111]
		Higher frequency of the XX genotype in the endurance athletes	[17,112]
	Power sports	Significantly lower frequencies of the XX genotype, and higher frequency of the RR genotype, compared to the control groups	[16,17,100,113,114]
	Soccer	Significantly higher proportion of the RR genotype than the control group	[115]
	Swimming	No significance reported	[95,116]
	Running (short distance)	No significance reported	[94,95,115]
		No significantly less XX genotype in sprinters. Increased frequency of RR and R allele in elite sprinters compared to control group	[16,21,97,112]
	Volleyball	No significance reported	[95,117]
*ADRB1*	Endurance sports	C allele is associated with increased VO_2_max, exercise time, and exhaustion. G allele is associated with decreased VO_2_max	[23,74,118]
*PPARG-C1A*	Endurance sports	Endurance athletes have a higher proportion of GG genotype, and a lower frequency of A allele	[21,22]
	Sprinting	GG genotype is associated with increased endurance ability and AA genotype may be associated with impaired aerobic capacity	[21,22]
